# 

**Published:** 1903-11-15

**Authors:** 


### CONTENTS.

Jonathan Taft, D.D.S., M.D. - - - - - - 541

First Molar Extraction During Childhood, and Its Relation to Occlusion and Physiognomy, *By Milton T. Watson, D.D.S.* 547

Benefits of Dental Societies, *By W. A. Barber, D.D.S.* 561

International Dental Federation, (Circular to the Presidents of National Dental Societies) - - - - 565

The Staple Crown, - - *By Dr. F. L. Marshall* 568

Immediate Root Filling, - *By O. E. Inglis, D.D.S.* 570

Press Comments on the Death of Dr. Taft - - - 575

Editorial, - - - - - - - - - 581

Bibliography, - - - - - - - - 583

Announcements, - - - - - - - - 584

Obituary, - - - - - - - - - 586

Indents, - - - - - - - - - 588

